# Monochromatic X-Ray Induced Novel Synthesis of Plasmonic Nanostructure for Photovoltaic Application

**DOI:** 10.1038/srep22394

**Published:** 2016-04-20

**Authors:** Amardeep Bharti, Richa Bhardwaj, Ashish K. Agrawal, Navdeep Goyal, Sanjeev Gautam

**Affiliations:** 1 Department of Physics, Panjab University, Chandigarh-160014, INDIA; 2 Neutron and X-ray Physics Division, Bhabha Atomic Research Center, Mumbai-400085, INDIA; 3 Dr. S.S. Bhatnagar University Institute of Chemical Engineering & Technology, Panjab University, Chandigarh-160014, INDIA

## Abstract

It has been universally delineated that the plasmonic metal nanoparticles can enhance the efficiency of photovoltaic cell by increasing the probability of energetic solar photons capturing phenomena using localized surface plasmonic resonance response. In this paper, we developed a novel *in-situ* simple approach to synthesize noble plasmonic silver nanoparticles (AgNP) from aqueous poly-vinyl-pyrrolidone solution of metal salt using radiolysis of water via synchrotron monochromatic X-ray irradiation without any chemical reducing agent. X-ray irradiation of water produces hydrated electrons 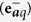, superoxide 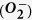 and atom radicals 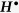, which triggers the reaction and reduces metal salt. X-ray radiolysis based synthesis provides the control over the reaction and prevent the formation of secondary products as occurs in case of chemical reduction route. In the previous studies, synchrotron “white” X-rays had been examined for the synthesis of metal nanoparticles, but that technique limits only upto the material synthesis while in this work we explored the role of “monochromatic” X-rays for the production of bulk amount of nanoparticles which would also provide the feasibility of *in-situ* characterization. Transmission electron micrographs show that the synthesized AgNP appears spherical with diameter of 2–6 nm and is in agreement with the size estimation from uv-vis spectra by “Mie theory”.

Plasmonic metal nanoparticles are of great interest in the field of green energy especially solar cell industry^1^. They build the foundation for enhanced light trapping in the device via their unique localized surface plasmon resonance response (LSPR) which could be easily tuned by optimising the size and shape of the nanoparticles^2^,^3^,^4^. For the application of solar cell, material required should be of high purity grade without any secondary product contamination. Numerous research activities have been reported to attempt these nanoparticles by chemical route in liquid^5^, gas phase^6^ and under high vacuum environment^7^. But most of the methods limit to control the reaction rate and the formation of secondary products. Radiation induced radiolysis synthesis overcome this problem and provides simple physico-chemical reaction, control over the reaction rate without any contamination under room temperature and atmospheric pressure^8^,^9^. Proton^10^, electron^11^, gamma^12^, and X-ray beams^13^ are the suitable irradiants to induce the reaction by the action of radiolysis of water, results in the generation of hydrated electrons 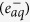 which plays the role of strong reducing agent towards metal ions^14^. Several attempts has been imparted to synthesize the metal nanoparticles by these radiation induced techniques, out of which gamma rays provide the low polydispersity product but lags behind due to their constrain over the safety concerns, irradiation time and *in-situ* characterization. On the other hand X-rays resolve these issues, provides longer irradiation time (as long as required), *in-situ* characterization^13^ and are widely available at laboratory and synchrotron light source.

Synchrotron light source provides the X-rays of variable energy with fine control over the wavelength which could be optimized for the desired application. X-ray energy is efficient to trigger the reaction by dosimeteric based radiolysis process, as it commands the reaction rate mechanism and provides the facility to investigate the element specific electronic structural properties^15^. Many research groups are indulged to use the synchrotron X-rays for the formation of metallic nanoparticles. Neal N. Cheng^16^ reported the chemical enhancement by NPs under the irradiation of X-rays. X-ray enhance the reaction yield resulting in the increased chemical properties. Yung-Chin Yang^17^ and Kuan-Nan Lin^18^ performed the synthesis of gold nanoparticles by controlling their size and shape using irradiation of synchrotron “white” X-ray beam. Qing Ma^19^ successfully achieved the formation of metallic pattern/structure on the substrate which would lead to next generation of electronics. One pot synthesis protocol for metallic (AuPt, AuNi) nanocomposites has been attained using synchrotron radiation by Cheng-Liang Wang^20^ and Chong-Cook Kim^21^. All such experiments were performed under synchrotron “white” X-ray beam, while S. Remita reported the synthesis of Ag-nanoparticles with highly focused monochromatic X-ray along with *in-situ* UV-vis spectroscopy as well as SAXS measurements^13^. We achieved the desired task with monochromatic, unfocused, and low integrated flux density X-ray beam that allows the formation of metallic nanoparticle in bulk quantity. Since the experiment is successfully conducted at much lower flux as compared to previous monochromatic focused beam experiments by S. Remita^13^, this opens the possibility of synthesis of plasmonic nanoparticles with high power laboratory based X-ray sources (LXS) thereby making it feasible to wide range of users from research and industry. Although “white” X-rays take lesser time in the process of synthesizing the nanoparticles than “monochromatic” X-rays, but still it dominate the white-beam because of its unique feasibility to provide the *in-situ* material characterization. X-ray techniques based *in-situ* characterization provides the possibility of investigating average particle size using SAXS^13^, electronic structure and chemical bonding using NEXAFS/EXAFS^22^,^23^, topology/morphology using nano-resolution imaging^24^ and crystalline structure using X-ray diffraction. This feature of monochromatic beam makes our method novel for the production of bulk amount of plasmonic nanoparticles along-with online optical & structural characterization and opens the gateway of new era for plasmonic research. This will also be helpful in controlling and tuning the properties of synthesized plasmonic nanoparticles through optimized irradiation dose and X-ray energy. Therefore synchrotron radiation based synthesis, though being expensive, is the only way to investigate the real-time electronic structure evolution with the growing size of nanoparticles, which is still a mystery. In this research work, we have developed a novel *in-situ* synthesis protocol for plasmonic silver nanoparticles (AgNP) from aqueous poly-vinyl-pyrrolidone (PVP) solution of metal-salt using synchrotron monochromatic x-ray without any chemical reducing agent, to make a better control over size and shape by optimizing the energy and exposure time. The effect of monochromatic beam energy at constant exposed radiation dose on structural properties of nano-particles formed is also studied.

## Methods

Silver nitrate and PVP of high purity grade were purchased from Sigma Aldrich and used as received without any further purification. Silver nitrate and PVP were dissolved in ultra-high pure water (Millipore) at 1:1 by weight. In these conditions, 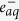 and 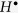 are formed due to X-ray induced radiolysis of water that act as reducing agents towards metal-ions resulting in the formation of plasmonic metal nanoparticles. An appropriate irradiation cell 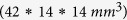 of polypropylene with kapton cavity has been designed for this experiment.

**Figure 1:**
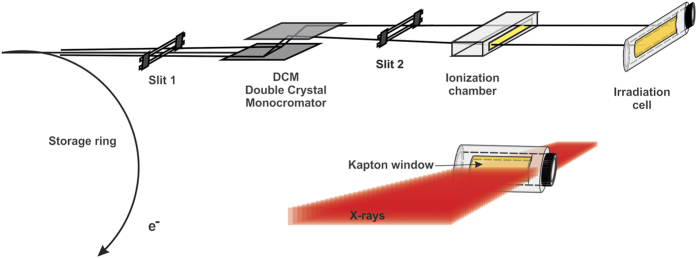
Optical layout of X-ray beamline at BL-04, Indus-2, INDIA.

Irradiation was carried out using unfocused monochromatic X-rays beam of energy 20 keV and cross-section area 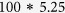 mm^2^ at BL-04(imaging beamline, 2.5 GeV storage ring energy and 135 mA maximum current), Indus-2, RRCAT (Indore) INDIA. The photon flux 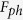 was 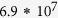 ph/s at energy 20 keV 25. Optical layout of BL-04 beamline is shown in the Fig. 1. The expression for integrated dose (Gy) imparted in the sample in such radiation induced experiments was derived as


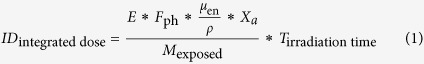


where *E *= Beam energy, *F*_*ph*_ = Photon flux density, 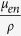 = mass energy-absorption coefficient (% absorption), 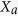 = Beam cross-sectional area, 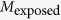 = integrated sample mass exposed to the radiation (exposed cell parameters × sample density), and *T* = total irradiation time. If the 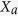 lies inside the irradiated cell parameters, the above equation may be written in a simple form:


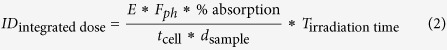


where 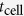 = cell thickness and 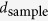 = sample density. Accordingly the energy transferred to the sample is 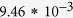 Joules/s and the integrated dose for the irradiation time of 01:50 hours comes out to be 62.435 Gy.

## Results and Discussions

X-ray irradiation of water produces hydrated electrons 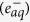, superoxide 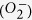 and atom radicals 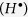, which triggers the reaction by reducing metallic-ions to zero valence silver 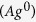 as below^26^:


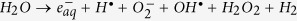



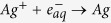



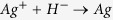



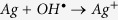


**Figure 2:**
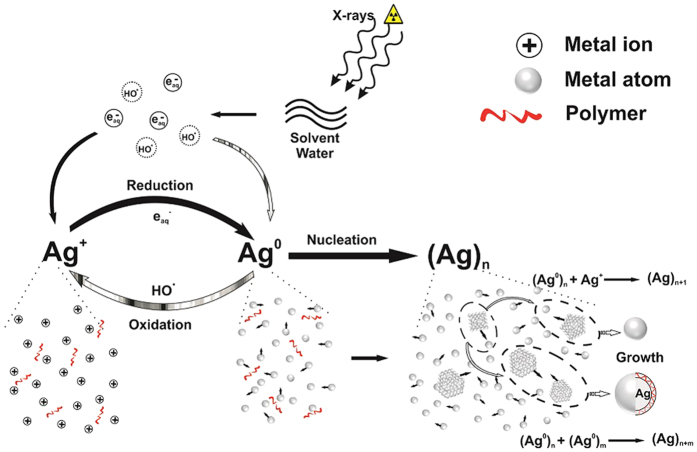
Scheme of metal-ion reduction in solution by X-ray irradiation.

**Figure 3:**
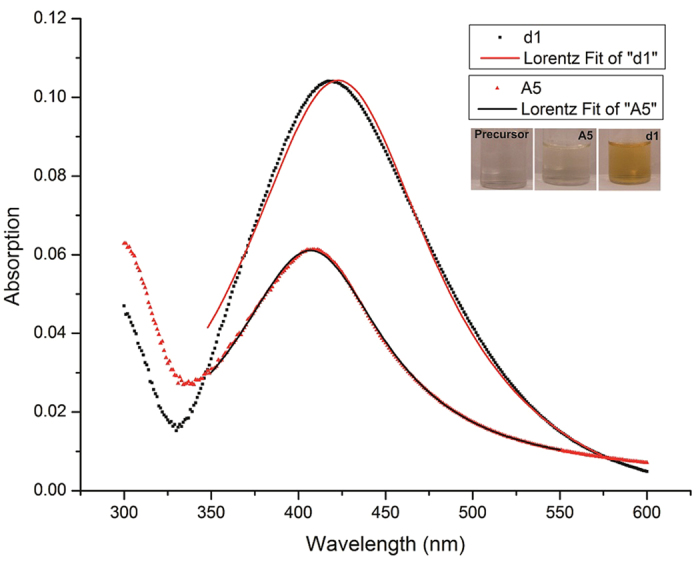
UV–Visible spectra of sample without (d1), and with scavenger (A5).

As the zero valent Ag-atoms are formed, particle growth begins to build nanostructure via nucleation process. 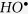 might cause the oxidation of 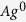 to 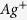, but the probability of oxidation is rare in comparison to reduction because of the large amount of 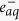, 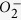 and 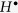 reducing agents than 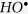. The two major events has been illustrated which are responsible for the formation of small and bigger particles as shown in Fig. 2. Number of 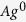 coalesce to form the nanostructure 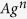, which further interacts with the 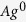 atom and synthesize the AgNP. If 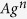 interact with 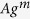 nanostructure, it results in the growth 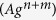 of AgNP with larger size. The colour phase transition from transparent white to yellow during the X-rays irradiation is attributed to the LSPR^27^ of AgNP and is the first confirmation towards the formation of nanoparticles. After the irradiation, product solution with appropriate dilution was characterized by the UV-Visible photo-spectrometer (JASCO V-630, USA). The UV-visible spectra of product sample after irradiation of aqueous solution of AgNO_3_ + PVP (d1) and AgNO_3 _+ PVP + isopropanol (A5) along-with the fitted curve is shown in the Fig. 3. The absorption band (422 and 406 nm) of both the samples lies in the characteristic range^27^ (380–430 nm) of AgNP and confirmed the formation of same.

**Figure 4:**
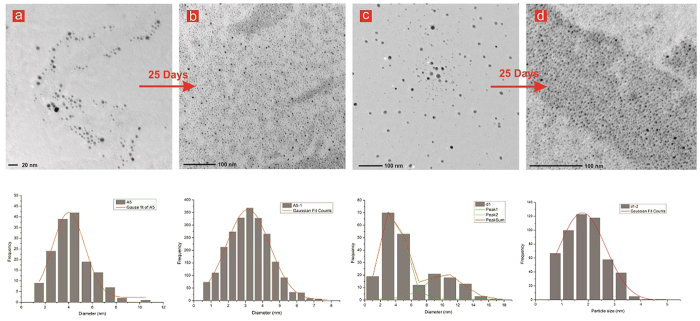
Transmission electron micrographs of sample A5 (a), repeated after 25 days (b), and similarly sample d1 is shown in (c,d) respectively along with their size histogram.

**Figure 5:**
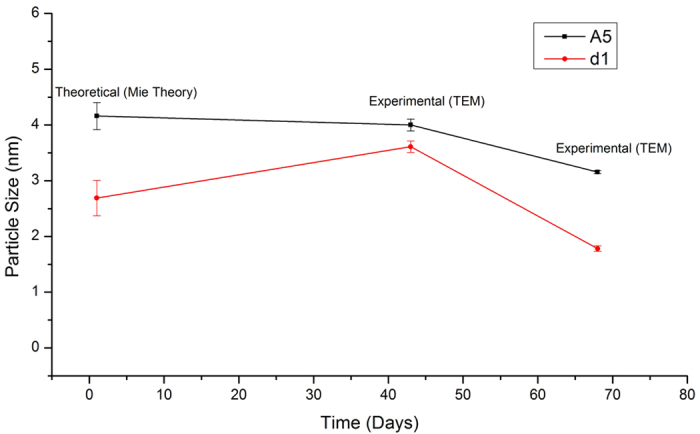
Size stability of the synthesized nanoparticle via X-ray irradiation.

**Figure 6:**
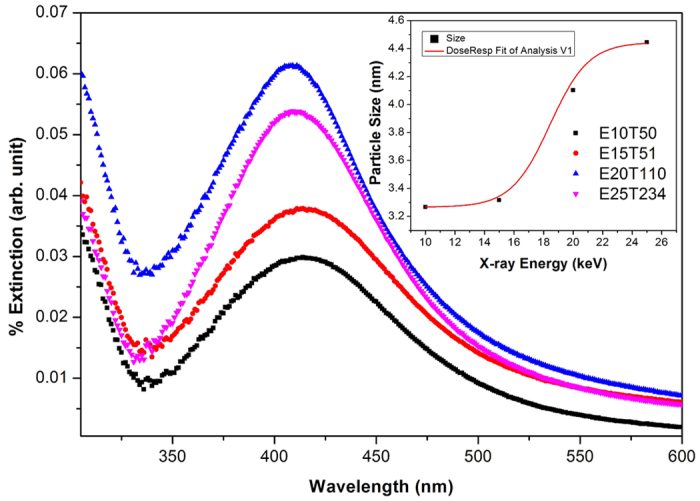
UV-Visible spectra of samples irradiated with different X-ray energies at constant irradiation dose. (Particle size evolution with energy-time product variation (inset)).

In the previous studies, isopropanol or ethanol had been introduced as a 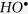 scavenger to avoid the oxidation of metal nanoparticles^13^. In our study, we have performed the experiment with and without the presence of this scavenger and investigate the actual mechnism. We did not observe any oxidation, which might be due to the presence of PVP that has enough capability to cap the particles and prevent them from the oxidation. Hence the presence of isopropanol/ethanol not only scavenges from oxidation, but also produces the reducing agent which reduce the size of nanoparticles. The centroid (peak-position) and the broadening (FWHM) of UV-vis spectra of AgNP depend upon the particle size, shape, and surface charge^28^. We have fitted/simulated the data according to the extended version of “Mie-Theory”^28^ to estimate the size of nanoparticles so formed. According to Mie-theory, particle size of samples d1 and A5 with absorption centroid at 422 and 406 nm, band width of 135.48 and 105.17 nm comes out to be 2.69 and 4.16 nm respectively (Table 1). Figure 4 shows the TEM micrographs of both the samples along-with the histograms. TEM micrographs were investigated by the imageJ package and data is fitted using Gaussian function. The calculated average size of NP for the sample d1 and A5 are 3.61 and 4.00 nm respectively. The histogram shows that the maximum intensity of the nanoparticles present in the solution is in agreement with the size estimation by extended version of Mie-theory (Table 1). To further check the self-lifetime of synthesized plasmonic nanoparticles, TEM measurements were repeated after the time span of 25 days and it is clear that there is no coagulation of nanoparticles as shown in Fig. 4. Synthesized nanoparticles have ultrahigh stability and retain the same size and shape profile as of freshly synthesized even after the age of 68 days (Table 1), as shown in the Fig. 5. A small decrement as observed in the average particle size is because of centrifugation of the samples, result in the highly concentrated product sample (clearly revealed in Fig. 4) and lead to collect the smaller particles also by TEM. In addition to this, the role of X-ray energy has been investigated by exposing the sample to different energies at constant irradiation dose. As the X-ray energy increases, the sample becomes more transparent to corresponding beam resulting in loss of irradiation dose. So the energy-time product has been set to keep the irradiation dose constant. Figure 6 shows the UV-Vis spectra of samples irradiated at different energies reveals that high X-ray energy produces the “hot” hydrated electron (although the dose is constant), which reduces the metal salt with inflate speed resulting in enhancement of particle size. The process of generation of “hot” 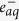 is dependent on X-rays energy, flux and sample holder size. The particle size and concentration is of utmost level at 20 keV in case of our sample holder dimensions. If, one selects a very thin sample holder then even low energy X-rays could lead to the formation of metallic nanoparticles.

The presented results shows the novel synthesis protocol for the production of bulk amount of AgNP under the irradiation of synchrotron monochromatic X-rays. This work also leads to real-time characterization of material which would help to understand the process of crystal growth phenomena and the evolution of electronic structure. This experimental technique delivers the scope for growing the nanoparticles with desired particle size, plasmonic and electronic properties that will definitely enhance the quality of photovoltaic cell. The particle size and reaction rate could be controlled by optimizing the radiation beam energy and dose for the production of high purity grade plasmonic metal nanoparticles. TEM report shows that the synthesized nanomaterial is more polydisperse in comparison with one obtained by the gamma ray irradiation. This is possibly because of the decrease in the radiation penetration depth in the sample due to the formation of nanoparticles by the time. And the second reason is due to the decay of beam current over the time results in decreasing the photon flux. To cover these issues, we plan to fabricate the irradiation cell with circular kepton cavity and uniform rotating mechanism.

## Additional Information

**How to cite this article**: Bharti, A. *et al.* Monochromatic X-Ray Induced Novel Synthesis of Plasmonic Nanostructure for Photovoltaic Application. *Sci. Rep.* **6**, 22394; doi: 10.1038/srep22394 (2016).

## Figures and Tables

**Table 1:** Results of size estimation by extended version of “Mie theory” and “Electron micrographs”.

Sample name	Centroid (nm)	Band Width (nm)	Estimated Size by“Mie Theory” (nm)	Experimental (TEM) predictedAverage Size (nm)
A5	406	105.17	4.16	4.00
A5	–	After one monthinterval	–	3.16
d1	422	135.48	2.69	3.61
d1	–	After one monthinterval	–	1.78
